# Venous bullet embolism to the right ventricle: Case report and review of management

**DOI:** 10.1002/ccr3.3284

**Published:** 2020-12-24

**Authors:** Luke Henderson, Ashley Wachsman, Joanna Chikwe, Fardad Esmailian

**Affiliations:** ^1^ Department of Cardiothoracic Surgery Smidt Heart Institute Cedars‐Sinai Medical Center Los Angeles CA USA; ^2^ Department of Radiology Cedars‐Sinai Medical Center Los Angeles CA USA

**Keywords:** cardiothoracic surgery, general surgery, vascular surgery

## Abstract

Intravascular missile emboli to the right heart should be retrieved surgically if the risk of surgical complication due to sternotomy and cardiotomy is low. Endovascular retrieval is another possible method of extraction to be considered.

## Graphical abstract

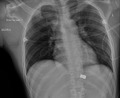

## 1 INTRODUCTION

Intravascular missile embolism, or bullet embolism, is a well‐known yet uncommonly encountered entity amid civilian trauma. This is despite the relative high rate of firearm‐related trauma in the United States.^1^ According to the American College of Surgeons’ National Trauma Data Bank, 4.2% of trauma admission are due to firearm‐related injuries.^2^, ^3^ Injury to the vascular system is a common finding in this group. In patients with gunshot wounds in proximity to peripheral vessels, 10% were found to have arterial injuries.^4^ Despite the frequency of firearm‐related vascular trauma in American trauma centers, intravascular bullet embolism is rare. The number of cases reported in the literature over the last 30 years suggests a rate of approximately 10 cases per year in the United States.^5^ The true incidence may be significantly higher.

In cases of bullet embolism, there is often limited time for clinical decision making. It is important for practitioners to be familiar with the features of intravascular bullet embolism and the principles of its management in advance. We present a case of a venous bullet embolism to the right ventricle of the heart following a gunshot wound to the right shoulder. Below, we will review the features of intravascular missile embolism and strategies for the management of right‐sided intracardiac missile emboli.

## 2 CASE REPORT

A 26‐year‐old man presented to the trauma resuscitation unit at Cedars‐Sinai Medical Center after a single gunshot wound. He was reportedly approached by an unknown male assailant and shot in the back while attempting to flee from the gunman at 9:55 am. He arrived to the trauma center 20 minutes later at 10:15 am. On arrival, the patient had no respiratory distress, with oxygen saturation of 98% on room air with respiratory rate of 18. His circulation was intact with a heart rate of 82 and blood pressure of 138/93. He was alert and oriented, moving all extremities, with no finding of neurological deficit.

On examination, he was found to have a single gunshot wound in the right upper back overlying the scapula. A slight fullness of the right upper chest and supraclavicular fossa suggested a hematoma. Breath sounds were intact and equal bilaterally. Radial pulses were easily palpable, and equal bilaterally. Jugular venous distention and pulses paradoxus were absent. No other traumatic injury was found on his secondary survey examination.

**Figure 1:**
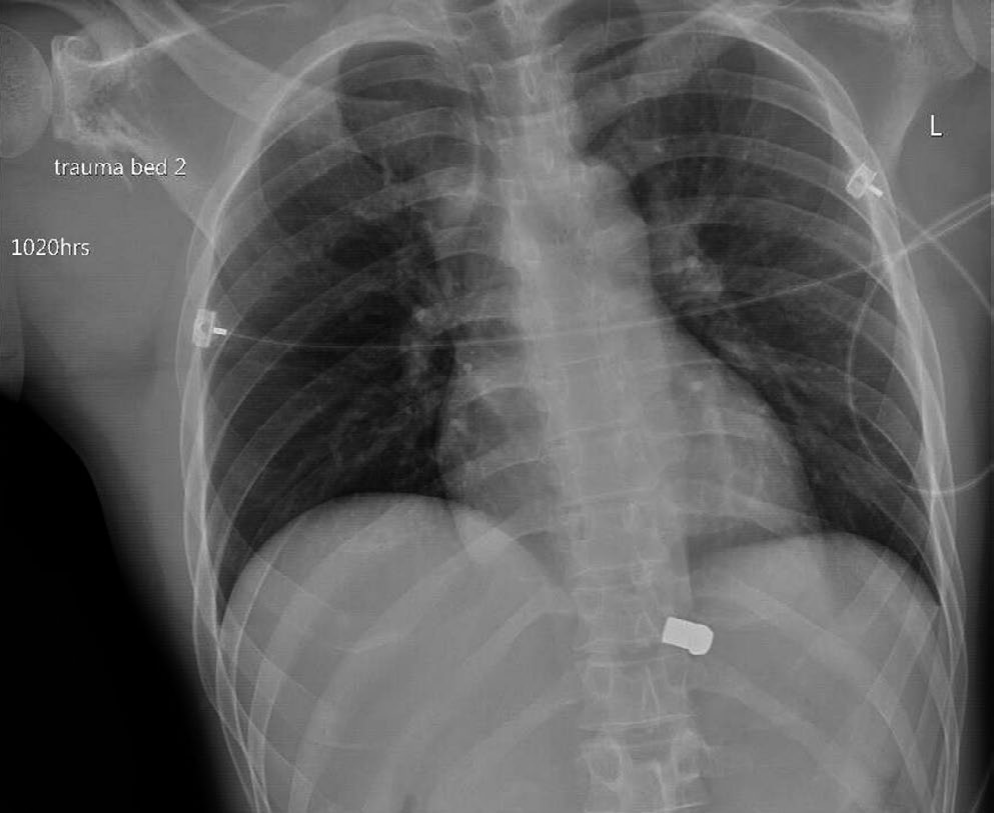
Initial chest X‐ray obtained in the trauma bay

A chest X‐ray showed a bullet projected over the spine at the level of the diaphragm (Figure 1). A comminuted right scapula fracture was seen. No pneumothorax, pleural effusion, or abnormality of the cardiac silhouette was seen.

**Figure 2:**
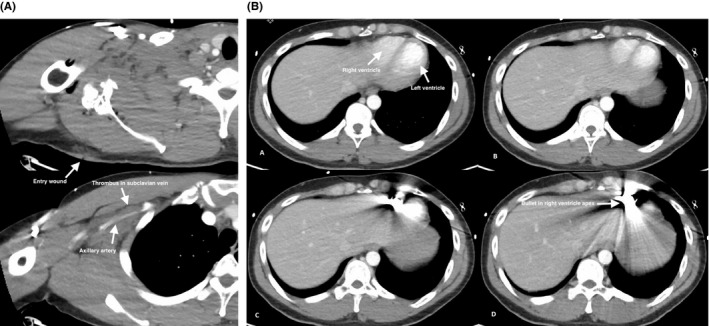
A, Computed tomography scan showing the right shoulder. Upper frame shows posterior gunshot wound and trajectory of the bullet through the glenoid of the scapula. Lower frame shows the axillary artery contrasted and the subclavian vein with a filling defect just anterior to the artery. B, Frames A‐D are sequential cuts, cephalad to caudad, showing the right and left ventricles clearly with a bullet in the right ventricle apex

The patient remained hemodynamically stable, and a contrasted computed tomography scan of the chest abdomen and pelvis was obtained which showed a right posterior shoulder entry point with blast injury involving the glenoid neck, and a nondisplaced fracture of the right clavicle. Extravasation of contrast was seen from the proximal right axillary artery, and a filling defect was seen in the right subclavian vein suggesting a thrombus. The bullet was located in the right ventricle of the heart. No injuries to the heart, lungs, or chest structures were found (Figure 2).

Cardiac surgery was consulted by the trauma surgery team. After seeing the patient, a bedside transthoracic echocardiogram was obtained which confirmed the location of the bullet in the right ventricle. After discussing treatment options with the trauma service and the patient, we decided to proceed urgently to the operating room for sternotomy and removal of the foreign object from his ventricle. Vascular surgery was also consulted to evaluate his axillary vein injury, and a noninterventional approach was preferred at the time.

**Figure 3:**
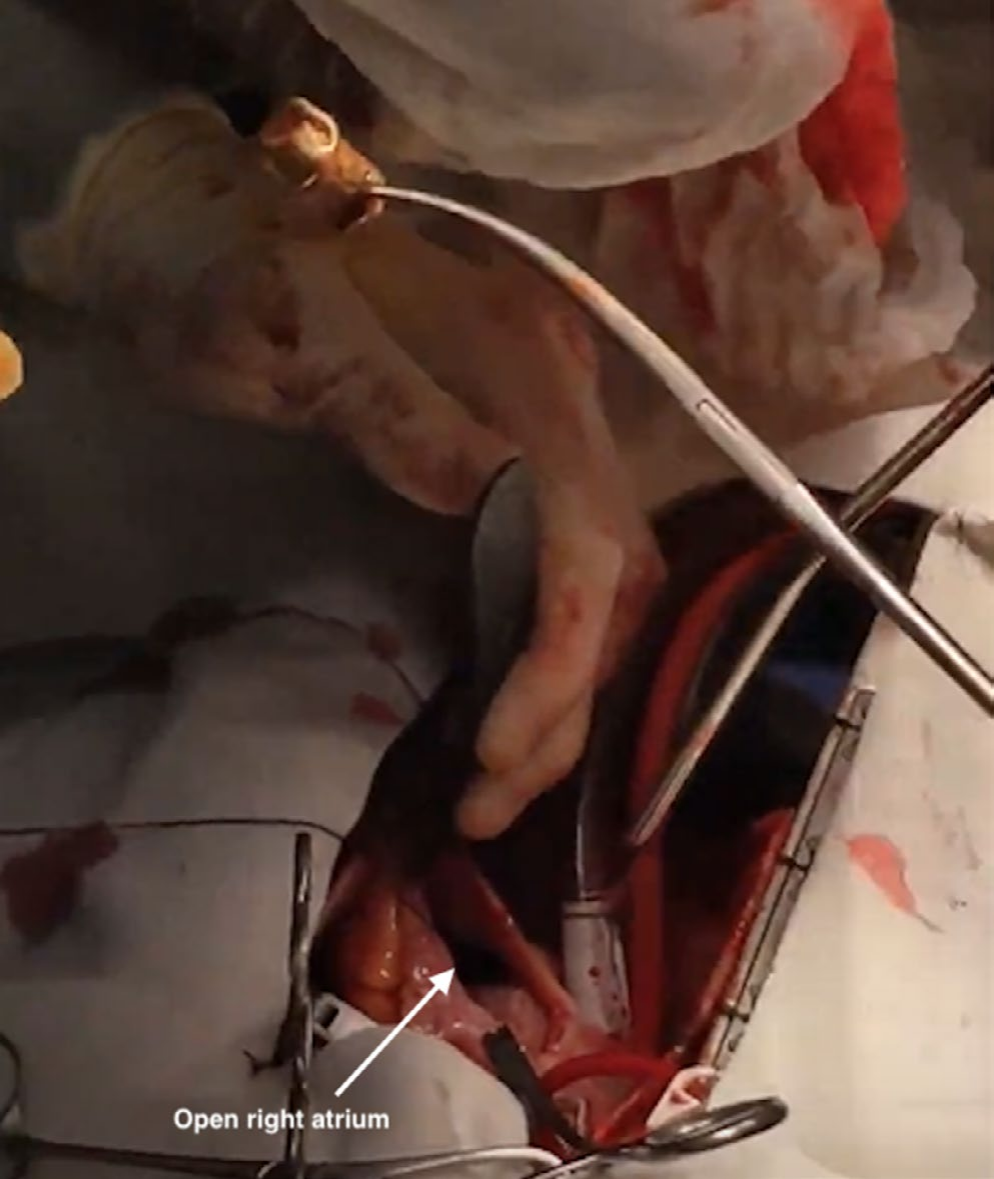
View of the bullet after removal from the right ventricle. The open right atrium is seen below

**Figure 4:**
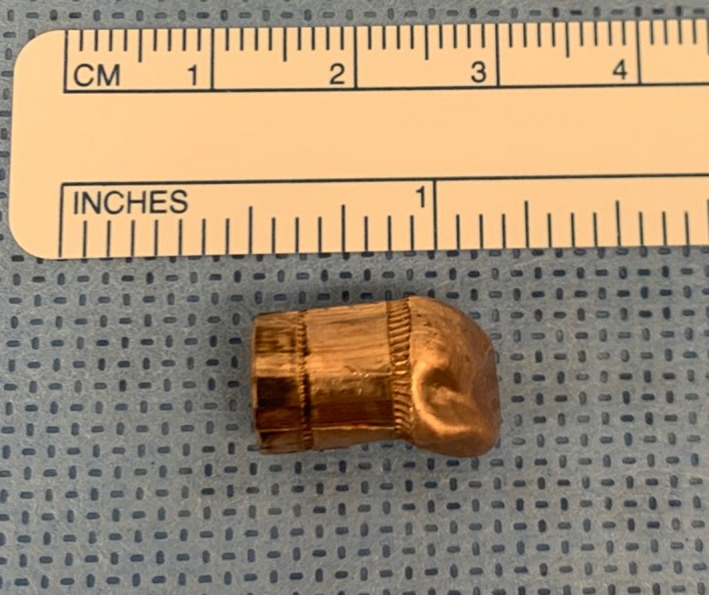
Deformed but intact, full metal jacketed handgun round. The diameter at the base of the bullet measures 0.375 inches or 0.38 caliber. This round is most likely from a 0.38 caliber handgun

In the operating room, having arrived at 11:55 am, a right internal jugular central line and arterial monitoring line were placed. A transesophageal echocardiography probe was inserted, and the bullet was again found in the right ventricle, not freely moving (see video below). A sternotomy was performed, and the patient was cannulated in bicaval fashion using right‐angled cannulae in the superior and inferior venae cavae with umbilical tapes ensnaring both cavae. Once adequate activated clotting time was achieved, the patient was placed on cardiopulmonary bypass and the heart was arrested for better exposure of the right ventricular apex. A right atriotomy was made, and the right ventricle was explored through the tricuspid valve. The bullet was found at the apex of the right ventricle seated between two trabeculae. A long tonsil clamp was used to remove the bullet. No dissection was required. The bullet appeared to be a large‐caliber handgun round and was deformed at its tip, with no fragmentation or splintering (Figure 3). The base of the bullet measured 0.375 inches and the diameter of a bullet for a 0.38 caliber revolver (Figure 4). The right ventricle and tricuspid valve was normal appearing otherwise. Transesophageal echocardiography was used to confirm the absence of any other foreign body. The right atriotomy was closed, and the patient weaned from bypass. Pacing wires and chest tubes were placed. The patient’s chest was closed after hemostasis was achieved. He did not require blood transfusion, nor did he require inotropic support.

Postoperatively, he progressed well. His right upper extremity examination continued to show no neurological or vascular abnormality. Arterial ultrasound of the right upper extremity showed patency of the subclavian, axillary, brachial, ulnar, and radial arteries. A venous duplex ultrasound examination of the right upper extremity showed no thrombus in any of the visualized veins including the subclavian vein. His fractures were managed nonoperatively, and Lovenox was started on postoperative day 2 for venous thrombus prophylaxis. Chest tubes were removed on day 3. After physical and occupational rehabilitation evaluation, he was discharged on day 5 in good condition.

## 3 DISCUSSION

There are at least several hundred published reports of intravascular missile embolism over the last 100 years.^6^ The largest series comes from the Vietnam War, where 22 cases of intravascular emboli were found among 7500 cases of vascular system penetrating trauma (0.3%), with most involving explosive device fragments (0.3%).^7^ A more recent series from the Global War on Terror found four cases of intravascular fragment emboli from 346 patients with penetrating vascular system injuries.^8^

By comparison, in civilian trauma, most firearm‐related injuries are from lower‐velocity handguns. The lower kinetic energy of handgun projectiles may result in more cases of missiles arresting within blood vessels as opposed to the through‐and‐through injuries seen with high‐velocity rifle gunshot wounds.

Kuo et al^9^ reviewed 261 cases of civilian missile emboli over a 30‐year period from 1988 to 2018. They defined several key characteristics of this phenomenon. Young male patients were the most common demographic. The most common injury type was single gunshot wound (48%), with the chest being the most common entry point (39%). Venous missile emboli (56%) were more common than arterial emboli (27%). A possible explanation for this finding may be the thinner walled structure of veins compared to arteries, leading to higher rate of penetrance. Venous missiles embolize to the right heart or pulmonary arteries in 77%‐83% of cases.^9^, ^10^

A chest X‐ray with a bullet overlying the cardiac silhouette will raise suspicion of intracardiac or intrapericardial location. A bedside transthoracic echocardiogram should be performed to confirm whether or not the missile is intracardiac and whether the missile is freely mobile within the heart. Computed tomography scanning is useful in a stable patient, although metallic scatter may obscure the exact location of the projectile. Transesophageal echocardiography is the modality of choice with the highest accuracy and resolution for viewing a suspected intracardiac missile. Transesophageal echocardiography should be performed prior to any attempt at endovascular or surgical removal of a suspected intracardiac foreign body. This can be done on the operating room table prior to intervention. The heart should also be examined for intracardiac shunts which make right‐to‐left intracardiac embolization possible. The information gained is essential in planning the approach, cannulation setup, and location of cardiotomy in the case of surgical removal.

Acute clinical manifestation of retained cardiac missiles is tamponade, hemorrhage, valvular dysfunction, and intracardiac shunts due to septal injury. Late manifestations are missile embolus to the pulmonary arteries, endocarditis, dysrhythmia, pericarditis, stroke, erosion into coronary vessels, lead toxicity, and cardiac neurosis.^11^ Missiles within the right chambers of the heart may become entrapped within the endomyocardial trabeculations becoming encapsulated in fibrous tissue.^12^

Options for management include observation, endovascular retrieval, and surgical removal. Endovascular retrieval of intracardiac missile emboli has been successfully performed in several case reports. In a recent case, a 28‐year‐old man was readmitted with palpitations after gunshot wound.^13^ A bullet embolus was found in the right ventricle, affixed to the free wall. A transjugular approach was used with fluoroscopy guidance, and the bullet was removed percutaneously with no damage to the tricuspid valve. A reasonable approach is to attempt percutaneous removal via a small venous cut‐down onto the right internal jugular vein with the use of fluoroscopy in the OR. If endovascular retrieval fails, one may proceed with surgical exploration. The likelihood of success may depend on the location and positioning of the missile, with higher rates of success expected in the right atrium.

Surgical removal of intracardiac missile emboli has been the most commonly reported approach to managing these patients, but controversy exists based on the multiple reports of successful management with observation.^9^ Lundy et al^14^ reviewed cases of patients with right‐sided intracardiac missiles treated nonoperatively and found a complication rate of 13% (n = 16). Other authors have reported complication rates in the range of 20%‐30%.^9^ Hard indications for surgical removal include: proximity to a vital structure (coronary artery/vein or conduction system), entry into heart after contamination, wandering missile on imaging, dysrhythmia, intracardiac shunt, and hemodynamic significant valvular compromise. Soft indications for surgical removal included sepsis, fevers, concomitant mediastinal exploration, and inability to establish follow‐up for serial imaging.^14^

In our case, a large nonmobile right ventricular bullet embolus was found near the right ventricle apex. An endovascular approach for removal was rejected based on location and positioning as there was no way to get around the bullet. Conservative management would have been a reasonable option based on the location of the missile and the fact that it appeared seated in the RV trabeculations. The large size of the bullet was concerning to us. If it dislodged and embolized, a large branch of the pulmonary artery could be occluded causing respiratory complications including lung infarction. There was also a concern for myocardial complications associated with erosion. Serial imaging and follow‐up were also problematic in this young and uninsured trauma patient. These concerns, coupled with a projected low risk of major complication from surgical removal (1%‐2%), led us to the decision to remove the bullet surgically.

## 4 CONCLUSION

Although rare, surgeons practicing at busy trauma centers with high rates of penetrating trauma are likely to see an intravascular or intracardiac missile embolus. Management options include observation, endovascular, and surgical removal. In the absence of definitive data to suggest management guidelines, the management approach should be selected on a case‐by‐case basis taking into consideration the specific characteristics of the intravascular missile and the patient’s overall clinical picture. Risks of a major or minor complication following surgical or endovascular removal must be weighed against the risk of complications with conservative management.

## CONFLICT OF INTEREST

The authors declare that they have no conflict of interest.

## AUTHOR CONTRIBUTIONS

Luke Henderson DO: drafted the authorship. Luke Henderson DO, Ashley Wachsman MD, and Fardad Esmailian MD: drafted the revision. Joanna Chikwe MD, Fardad Esmailian MD, Ashley Wachsman MD, and Luke Henderson DO: involved in Final approval of draft to be published.

## ETHICAL APPROVAL STATEMENT

Published with the written consent of the patient.

## Supporting information

Video S1
